# BXD Recombinant Inbred Mice as a Model to Study Neurotoxicity

**DOI:** 10.3390/biom11121762

**Published:** 2021-11-25

**Authors:** Airton C. Martins, Caridad López-Granero, Beatriz Ferrer, Alexey A. Tinkov, Anatoly V. Skalny, Monica M. B. Paoliello, Michael Aschner

**Affiliations:** 1 Department of Molecular Pharmacology, Albert Einstein College of Medicine, Bronx, NY 10461, USA; airton.dacunhamartinsjunior@einsteinmed.org (A.C.M.); beatriz.ferrervillahoz@einsteinmed.org (B.F.); monibas2@gmail.com (M.M.B.P.); 2 Departamento de Psicología y Sociología, Campus Ciudad Escolar, Universidad de Zaragoza, 44003 Teruel, Spain; cgranero@unizar.es; 3 Laboratory of Molecular Dietetics, IM Sechenov First Moscow State Medical University, 119435 Moscow, Russia; tinkov.a.a@gmail.com; 4 Laboratory of Ecobiomonitoring and Quality Control, Yaroslavl State University, 150003 Yaroslavl, Russia; 5 World-Class Research Center “Digital Biodesign and Personalized Healthcare”, IM Sechenov First Moscow State Medical University (Sechenov University), 119435 Moscow, Russia; skalnylab@gmail.com; 6 Department of Bioelementology, KG Razumovsky Moscow State University of Technologies and Management, 109004 Moscow, Russia

**Keywords:** genetic, alcohol, metals, cocaine, pesticides

## Abstract

BXD recombinant inbred (RI) lines represent a genetic reference population derived from a cross between C57BL/6J mice (B6) and DBA/2J mice (D2), which through meiotic recombination events possesses recombinant chromosomes containing B6 or D2 haplotype segments. The quantitative trait loci (QTLs) are the locations of segregating genetic polymorphisms and are fundamental to understanding genetic diversity in human disease susceptibility and severity. QTL mapping represents the typical approach for identifying naturally occurring polymorphisms that influence complex phenotypes. In this process, genotypic values at markers of known genomic locations are associated with phenotypic values measured in a segregating population. Indeed, BXD RI strains provide a powerful tool to study neurotoxicity induced by different substances. In this review, we describe the use of BXD RI lines to understand the underlying mechanisms of neurotoxicity in response to ethanol and cocaine, as well as metals and pesticide exposures.

## 1. Introduction

The development of an optimal experimental design requires the selection of an adequate animal model. The challenges in genetic studies mandate experimental platforms that afford complex models with the inherent ability to study the interaction between genes and environment in the context of phenotypic features. The BXD recombinant inbred (RI) murine cohort has been beneficial in advancing the discipline of neurotoxicity, among many others. The BXD RI lines represent a genetic reference population derived from a cross between C57BL/6J mice and DBA/2J mice using a strategy of advanced intercrosses [1,2]. The BXD RI mice were created by crossing two inbred parental strains repeatedly (for >20 generations) to ensure that they were at least 99% inbred [2]; therefore, it is noteworthy that all animals were derived from a single pair of parents.

The BXD RI murine family consists of approximately 150 strains with greater than 6 million sequence variants, a level similar to the human cohorts that are being studied in genome-wide association studies (GWASs). Therefore, the BXD RI model has been considered adequate for the analysis of the genetic variability in the context of human disease etiology [1,3]. As these strains are inbred, the same genome can be studied repeatedly, providing an optimal model to investigate disease models [4]. Currently, the BXD RI family has been extended to 150 inbred strains [1].

Originally, the BXD RI mice were used to investigate the toxicologic effect of cadmium (Heiniger, n.d.). In this study, the authors addressed genetic susceptibility in the BXD RI mice related to necrotic damage. In these initial experiments, the field of genetics was reduced to Mendelian traits [1]. More recently, thanks to progress in genomic sequencing [5], the BXD RI mice were used to map complex traits. This advance has led to the use of BXD RI mice in addressing neurotoxicology and neurodegeneration [6,7]. Specifically, the relevance of the BXD RI family has been extended to the analysis of the genetics of behavioral phenotypes, including alcohol and drug addiction [8,9,10,11,12,13,14], neurodegenerative processes [4,6,15,16,17], heavy metals and pesticides [3,18,19], and changes related to the degeneration of the visual system [20,21,22].

Combined, these studies have proven the advantage afforded by the BXD RI family in querying the genetic determinants of neurotoxicity. Our research group has assessed BXD RI mice in the context of social preference, anxiety and depression-associated behaviors, along with changes in the inflammatory system in the brain cortex [6]. In addition, we reported on the utility of BXD RI mice as a model for innate inflammatory responses in distinct brain regions, such as the hippocampus, striatum, and cerebellum [7]. Our studies highlighted the need to identify an adequate animal model based on the neurotoxicology and behavioral objectives, with specific BXD RI mice strains providing unique, innate characteristics.

It is also noteworthy that the recombinant inbred strategy is not specific to mice in a laboratory setting. In the natural environment of rodents, consanguinity is frequent in the wild state [23].

In the present review, we leverage the information of available studies which take advantage of the BXD RI mice cohort to highlight the great utility of these mice in studying innate inflammatory responses linked to behavioral alterations in the context of neurotoxicology. We discuss the potential for the BXD RI family to advance the mechanistic understanding of neurotoxicity in response to ethanol and drugs of abuse, as well as metals and pesticide exposures.

## 2. Ethanol

The consumption of ethanol is an important health concern, and its abuse represents a risk factor for several diseases, such as liver cirrhosis, hypertension, and numerous cancers [24]. Societal and cultural environmental factors contribute to the consumption of ethanol, as well as genetic factors that predispose one to alcoholism [25]. In this regard, studies using BXD recombinant inbred (RI) mice have been profitably carried out to investigate and identify genes that are associated with alcohol sensitivity and a predisposition to alcoholism.

Pioneering research using BXD RI mice was carried out by Crabbe et al. in 1983, which assessed the responsiveness to ethanol and demonstrated that distinct strains are differentially sensitive to ethanol withdrawal severity [26]. Another study reported on the severity of acute withdrawal from ethanol in mice from the inbred strains C57BL/6J and DBA/2J, as well as 19 of their RI (BXD RI) strains. The authors used handling-induced convulsion (HIC) as a score to measure the severity of ethanol (EtOH)withdrawal. The study demonstrated that withdrawal HIC is related to a cluster of genes (Scn1, Scn2, Scn3) that encode for voltage-sensitive sodium channel protein, as well as the Gad1 gene which encodes for the synthesis of glutamic acid decarboxylase [27]. The same research group used BXD RI mice to identify quantitative trait loci (QTLs) associated with the sensitivity and tolerance magnitude to ethanol-induced hypothermia. Approximately 800 mapped polymorphic genetic markers were associated with sensitivity and/or tolerance to ethanol, and QTL analyses identified potential specific candidate genes that may contribute to ethanol-induced hypothermia [28].

In addition, studies with the BXD RI cohort have identified chromosomal regions containing genes that contribute to ethanol craving, as well as behavioral and locomotor responses. Locomotor activity was used in these studies to assess the initial sensitivity to alcohol [29,30]. Interestingly, Phillips et al. (1995) studied male BXD RI strains upon acute and repeated dosing with 2 g/kg ethanol. The authors found that both acute and repeated treatments led to changes in the initial ethanol response, which was attributed to the sensitization of locomotor stimulation; QTL analyses demonstrated an association with basal locomotor activity and acute locomotor response on chromosomes 3, 4, 7, 11, 12, 13, 15, and 17 to initial ethanol response [31]. Likewise, a study with 20 BXD RI murine strains and C57BL/6J and DBA/2J progenitor strains exposed to 2 g/kg of ethanol evaluated ethanol’s ability to induce conditioned place preference, noting a significant association between ethanol-seeking behavior in BXD RI mice and genetic markers on chromosomes 4, 8, 9, 18 and 19 [32].

Moreover, locomotor activity induced by 1.5 g/kg ethanol was evaluated in a panel of 25 BXD RI strains, and QTL analyses identified and validated an association between ethanol and locomotor activity on chromosomes 1, 2, 4, and 6. An additional analysis using an intercross of male C57BL/6J X DBA/2J F2 animals confirmed the putative QTL on chromosome 2 [33]. Furthermore, the relationship between ethanol and susceptibility to incoordination effects was tested in an expanded panel of BXD RI mice to identify QTLs: QTLs for EtOH-induced locomotor activation on chromosomes 2 and 5 and a novel QTL for EtOH-induced motor incoordination on chromosome 7 were identified [34].

In evaluations of the genetic susceptibility to ethanol consumption and withdrawal, the BXD RI strains have been of particular interest, as C57BL/6J (B6) inbred mice are able to drink large amounts of ethanol voluntarily, while DBA/2J (D2) mice show improved withdrawal to acute ethanol. Hence, these strains show contrasting behavioral responses to ethanol [35]. Indeed, Browman and Crabbe performed QTL analyses in 25 BXD RI murine strains derived from a cross of C57BL/6J (B6) and DBA/2J (D2) progenitors. Male mice were injected with 4.1 g/kg ethanol and showed a loss of the righting reflex and ataxia, which correlated with ethanol sensibility. Interestingly, QTL analysis showed a difference in sensitivity among the strains, reflecting genetic variation where the alleles that decreased sensitivity to ataxia were observed in the B6 strain, while those alleles determining increased sensitivity to ataxia were observed in the D2 strain [36].

QTL analyses in DBA/2J (D2) and C57BL/6J (B6) mouse strains identified several chromosomal regions involved in risk for acute ethanol withdrawal and behavioral responses to ethanol [37,38]. However, the brain region(s) and circuit(s) by which these genes and their protein products influence the physiological dependence on alcohol have been poorly explored. Kozell et al. (2004), using D2 and B6 strains, administered ethanol (4 g/kg) and evaluated the numbers of c-Fos immunoreactive neurons in 26 different brain regions. c-Fos induction (neural activation) within the circuitry differed in terms of neural activation within the basal ganglia, suggesting the involvement of acute ethanol withdrawal and reflecting the distinct genetics of the basal ganglia circuity [39]. More recently, BXD RI mice derived from the C57BL/6J (B6) and DBA/2J (D2) inbred strains were used as a genetic approach to assess the relationship between acute ethanol effects and hippocampal Coenzyme Q expression since a previous study showed that *Coq7* could play a role in alcohol consumption [40]. It was reported that the acute ethanol-induced increase in the expression *Coq7* and 45 ethanol-related phenotypes, including six phenotypes related to conditioned taste aversion and ethanol preference [41].

## 3. Drugs of Abuse

The use of legal and illicit psychoactive substances represents an important public health concern. Drug addiction is characterized by the loss of control of drug intake, craving, and withdrawal [42]. Drugs of abuse can lead to serious health problems and impact socioeconomic and familial relationships. Several risk factors contribute to the initiation of and ongoing dependence on drugs of abuse. Environmental factors, as well as sex, age, peer influences, social support, stress, diet, and previous drug exposure, have all been implicated in influencing drug addiction [43,44]. Moreover, genetic factors contribute to an individual’s response to drugs of abuse from initiation to addiction. Therefore, studies aimed at identifying specific genes that are associated with drug addiction and dependence have been profitably carried out [45,46], as detailed below.

Cocaine abuse can lead to neurotoxicological effects, and continuous use is associated with drug-seeking behaviors and addiction. Genetic factors have been investigated for responsiveness to cocaine, dependence, and predisposition; genetic mouse models, such as the BXD RI cohort, have provided evidence for an underlying genetic role in cocaine-induced behavior, dependence, and withdrawal [47,48,49]. For example, gene(s) associated with cocaine-induced adverse effects, such as seizures, have been described. The first study that identified putative chromosomal loci associated with a cocaine-related phenotype was carried out by Miner and Marley (1995). The study found that after an intraperitoneal injection of 60 mg/kg cocaine in 26 BXD RI strains, a QTL analysis of cocaine seizure susceptibility showed a skewed, bimodal range, exhibiting nine provisional QTLs that reached the *p* < 0.05 level. A significant correlation clustered in two regions on chromosomes 6 and 12, correlating with the susceptibility to cocaine-induced seizures [50]. Likewise, BXD RI strains and an F_2_ population derived from the B6 and D2 progenitor strains were evaluated to determine genes that influence seizure susceptibility. The QTL analyses exhibited a significant correlation for three different chromosomes: one for clonic seizures on chromosome 9 (distal) and two for tonic seizures on chromosomes 14 (proximal to mid) and 15 (distal). Moreover, two other QTLs that reached (*p* < 0.0015) were associated with the susceptibility to cocaine-induced seizures on chromosomes 9 (proximal) and 15 (distal). Interestingly, the QTLs on chromosome 9 were sex-specific, where the effects on the phenotype were higher in females than in males [51].

Cocaine interacts with the dopamine transporter (DAT), blocking dopamine uptake and increasing its synaptic availability. Increased DAT expression has been reported in regions of the human brain of cocaine-abusing individuals [52,53]. Using BXD RI strains and progenitors, genes on proximal chromosome 19, which influence DAT expression, were mapped and correlated with DAT expression levels in 20 BXD RI strains in terms of behavior (locomotor response) and thermic responses (hypo- or hyperthermia). However, DAT expression was not correlated with other behaviors, such as rewards, voluntary consumption, and seizures [54].

It is well-established that activating and sensitizing the locomotor are important behavior assessments for acute cocaine response. Indeed, an association via QTL mapping and sensitization on chromosomes 1–10, 13, 15, 18, 19, and X were found. In addition, QTLs for locomotion were mapped to chromosomes 1, 3–6, 9, 12, 13, 18, and 19 and associated with cocaine response [55]. BXD RI strains and QTL analysis have also been combined with other tools, such as GeneWeaver software, to integrate and aggregate multiple studies of addiction genomics, which led to the identification and validation of *Rab3b* as a candidate gene for cocaine-induced locomotor activation [56]. Hence, using genetic analysis systems that integrate BXD RI animal models and new bioinformatics approaches are invaluable tools for investigating cocaine addiction.

## 4. Metals

BXD RI mice have been used in exploratory studies to understand trace metal accumulation in the brain [57,58]. Quantitative trait loci (QTL) and genetic correlational analysis in strains of the BXD RI panel have been used to advance the genetic mechanisms underlying the homeostasis of trace metals such as iron (Fe), copper (Cu), and Zinc (Zn) [18,59,60].

### 4.1. Iron

Iron is a trace element that can donate and accept electrons, therefore participating in multiple biological processes. It is essential for the normal functioning of the central nervous system (CNS), playing a role in processes such as neurotransmitter synthesis and myelin formation [61,62,63], to name a few. At the same time, an excess intracellular burden of iron, especially ferrous iron (Fe^2+^), is toxic and triggers the production of reactive oxygen species (ROS) [64,65]. Alterations in iron homeostasis are linked to numerous neurological diseases, such as Parkinson’s disease [66] and Alzheimer’s disease [67], whereas iron deficiency has been associated with Restless Legs Syndrome (RLS) [68] and cognitive impairment [69,70].

Iron regulation is complex and involves multiple genes. In humans, iron regulation shows wide individual differences [71]. To better understand the genetic causes of these differences and the failure to control iron homeostasis, the genetic reference population of BXD RI strains has been profitably studied.

Jones and colleagues, using genetic correlation and QTL analysis in a panel of 15 BXD/Ty RI strains, fed mice a diet containing 240 ppm of iron and identified 11 regions with a nucleotide polymorphism on chromosomes 1, 2, 5, 7, 9, 11, 13, 14, 17, and 18 in the ventral midbrain (VMB) [72]. The genetic correlation between liver and VMB iron content suggested that the central and peripheral iron regulation mechanisms are independent [72]. Similarly, a study using a panel of 22 BXD RI strains, fed a normal diet (240 ppm of iron) or an iron-deficient diet (3–5 ppm iron) for 120 days, identified the glial glutamate transporter 1 (GLT1) gene on chromosome 2 as a key regulator gene of iron homeostasis. Its expression correlated with the VMB iron content, and it was shown to be susceptible to iron deficiency [63].

Iron deficiency has been linked to altered dopamine function and the development of several neurological diseases, such as RLS [73,74]. However, the genetic mechanisms for iron–dopamine interactions have yet to be fully understood. BXD RI strains fed an iron-deficient diet for 100 days identified two new genes in the VMB which have been implicated in the iron–dopamine pathway. These genes were chemokine (C-X-C motif), ligand 12 (Cxcl2), and hemoglobin, beta adult chain 1 (Hbb-b1) [75]. Cxcl12 encodes a chemokine that activates the nigrostriatal dopamine system [76]. It is highly expressed in VMB, and its levels increased in mice fed an iron-deficient diet. The BXD 40 and 19 strains displayed the highest increase in Cxcl12 expression. Hbb-b1 is also activated under iron-deficient conditions. It encodes the beta chain of hemoglobin. Its main role is related to the oxygen transport in the blood. However, it is also expressed in dopamine neurons [77]. The overexpression of Hbb-b1 and Hba-a1 in mouse dopaminergic neuroblastoma cell line MN9D suggests a role for these genes in oxygen homeostasis, oxidative phosphorylation, and iron metabolism [77]. The BXD RI mice fed an iron-deficient diet showed an increased expression of Hbb-b1 in VMB. From the examined strains, the BXD 19 and 31 strains showed a higher up-regulation of Hbb-b1 when they were fed an iron-deficient diet [75].

In humans and rodents, iron levels fluctuate throughout the day in plasma and peripheral tissues [78]. Similarly, in an eight-strain BXD RI panel, brain-region-specific diurnal changes in iron concentration were identified. During the dark phase, iron levels were lower than in the light phase in females for the majority of the analyzed strains (BXD 14, 21, 31, 34, 40, and 42), whereas this difference was only significant in males in the BXD 21, 31, and 41 strains [60]. Similarly, studies with the BXD RI 40 strain demonstrated diurnal variations in brain iron content [79]. Iron deficiency resulted in a decrease in whole brain iron content. However, the decrease observed during the light phase in males and females fed a normal diet (containing 50 µg/g iron) disappeared in the BXD 40 fed an iron-deficient diet (3 µg/g iron) [79].

### 4.2. Copper

Copper is an essential trace metal for the development and function of the CNS. It acts as a cofactor for a variety of regulatory proteins and enzymes [80]. Alterations in copper regulation lead to neurological diseases. In Menkes disease, a genetic mutation in copper-transport protein ATPase 7 leads to a copper deficiency that promotes abnormal arborization of neurons, hypotonia, seizures, developmental delays, and death [81]. Copper excess is also deleterious for the normal function of the CNS. In Wilson’s disease, copper gradually accumulates in the brain causing motor dysfunction, emotional instability, psychotic episodes, depression, attention deficits, and sleep anomalies [82].

The analysis of a panel of 15 BXD RI strains revealed a significant QTL on chromosome 16 for copper regulation in the caudate-putamen (CP) of males, and a suggestive QTL on chromosome 12 in the CP and nucleus accumbens (NA) in females and on chromosomes 3, 13, and 17 in the prefrontal cortex (PFC) of males [18]. In the same study, copper was more concentrated in the VMB and PFC, whereas the NA showed a lesser accumulation of this metal [18]. Similarly, a study in the hippocampus using mice from 30 strains of the BXD/TY RI panel identified two QTL in chromosomes 14 and 9 strongly associated with copper homeostasis [59].

### 4.3. Zinc

Zinc is an essential trace metal and a cofactor for several proteins and enzymes. It also has an important role in protein folding [83]. In the brain, zinc accumulates in synaptic vesicles where it is co-released with glutamate [84]. It plays a neuromodulatory role, regulating excitability via NMDA and GABA-A receptors [84]. Zinc plays a critical role in many functions, including learning and memory [85]. Thus, zinc homeostasis is essential for brain function, and disruption in zinc levels is involved in neurological diseases. Zinc deficiency during childhood is associated with developmental disorders, mental retardation, and learning disabilities [86]. Alterations in zinc levels have also been shown to be associated with neurodegenerative diseases, such as Alzheimer’s disease and Parkinson’s disease [80,87,88,89].

QTL analysis in a 15 BXD/TY RI panel identified several chromosomal regions that influence zinc content in different brain regions. In PFC and VMB from females, a significant QTL area was found on distal chromosome 1. Another QTL region was found on chromosome 8 in the nucleus accumbens NA of females. In males, three different QTL were found in PFC on chromosomes 3, 13, and 17 [18]. Similarly, a study with 30 strains of the BXD/Ty RI panel identified a QTL on chromosome 9 that correlated with zinc content in the hippocampus [59].

## 5. Pesticides

The combination of high circulating levels of stress hormone (cortisol) [90] and exposure to irreversible acetylcholinesterase (AChE) inhibitors has been implicated in the etiology of Gulf War Illness (GWI), a medically unexplained chronic condition inherent to 25–32% of troops that served during the first Gulf War in 1991–1990 [91,92,93,94].

Exposure to a wide range of AChE inhibitors by the troops has been implicated, including pyridostigmine bromide, sarin, organophosphate and carbamates pesticides, as well as DEET (insect repellant, N,N-diethyl-meta-toluamide) [91,93,94]. The main symptoms of GWI are the result of neuroimmune activation and are similar to most of the characteristics of “disease behavior”, which comprises fatigue, musculoskeletal pain, cognitive dysfunction, chemical sensitivities, loss of memory, and insomnia [3,95]. However, heterogeneity in the symptoms of GWI has been observed, which may be explained by different exposures in addition to individual susceptibility to the exposures [94].

A study performed by Xu et al. (2020) used a genome-wide transcriptome approach in a genetically diverse population to more accurately model genetic changes that occurred to those persons deployed in the Persian Gulf. The study examined whole-genome RNA-seq in the prefrontal cortex of 30 BXD RI strains, plus the parental strains (C57BL/6J (B6) and DBA/2J (D2), aiming to identify common responses across strains, variants which mediate neuroinflammatory responses to the treatment, and the extent to which genetic differences alter neuro-morphological responses. The animals were divided into three treatment groups: the control group, DFP (diisopropyl fluorophosphate) group, and CORT (corticosterone) + DFP group. Although DFP is not used as a pesticide, it possesses anticholinesterase activity and has been used as a model organophosphate (OP) compound in mouse strain comparisons. Interestingly, a significant genetic effect in response to CORT + DFP treatment was observed, mirroring gene and protein expression changes seen in individuals with GWI. By using BXD RI strains, this study was able to identify genes and map loci influencing these outcomes [3].

Twenty-two different BXD strains plus C57BL/6J and DBA/2J progenitor strains were used in order to determine locomotor activity levels after acute exposure to the organophosphate compound paraoxon. This compound is the oxygen analog of the organophosphate insecticide parathion. The doses ranged between 0 and 1 mg/kg of paraoxon. The results showed that paraoxon treatment reduced locomotor activity in most but not all BXD strains, concluding that the magnitude of locomotor depression was dependent on the genotype [96].

## 6. Conclusions

Individual variation in response to several compounds and toxicants is due to a combination of environmental factors and genetic susceptibility. To understand how genetic variation may affect susceptibility, animal models have been developed and extensively used. The BXD RI model has emerged as an interesting and important model in biomedical, pharmacogenetic, and environmental toxicology studies. It affords an invaluable approach for screening for risk of addiction to legal and illegal drugs, susceptibility to intoxication by pesticides, and investigation on underlying homeostatic changes in response to exposure, to name a few. Table 1 summarizes the main findings in the studies presented in this review.

This model has been successfully applied, providing invaluable information on how distinct phenotypes can be mapped to chromosomal gene locations. The BXD RI lines have also permitted the identification of phenotypic differences in early drug sensitivity and candidate genes that may be linked to complex behavioral traits.

However, the limitations of the cohort must also be recognized. These include the fixation on common behavioral phenotypes, studies with just one sex, or those focusing on a small number of strains. Furthermore, the BXD RI model should be combined with new bioinformatic approaches to enable the identification of genetic markers responsible for trait variation and the covariation of susceptibility and behavior for different substances, xenobiotics, or essential metals.

## Figures and Tables

**Table 1:** An overview of the use of BXD RI strain in studies of the neurotoxicity in response to ethanol, cocaine, metals and pesticide exposures.

Substance	Dose	Mainly Finds	Reference
Ethanol	Inhalation of ethanol vapor for 3 days	Influence of gene on ethanol withdrawal severity	[26]
Ethanol	4 g/kg	A cluster of genes (Scn1, Scn2, Scn3) code for voltage-sensitive sodium channel proteins, genes candidates for affecting withdrawal ethanol	[27]
Ethanol	4 g/kg	800 mapped polymorphic genetic markers were associated with sensitivity and/or tolerance to ethanol	[28]
Ethanol	2 g/kg	QTL analyses identified significant marker associations with basal activity, acute locomotor response on chromosomes 3, 4, 7, 11, 12, 13, 15, 17	[31]
Ethanol	2 g/kg	Association between ethanol-seeking behavior and genetic markers on chromosomes 4, 8, 9, 18 and 19	[32]
Ethanol	1.5 g/kg	Association between ethanol and locomotor activity on chromosomes 1, 2, 4, and 6	[33]
Ethanol	2.25 g/kg	Ethanol-induced locomotor activation on chromosomes 2 and 5	[34]
Ethanol	4.1 g/kg	QTL analyses correlating strain means with allelic status at >1500 markers	[36]
Ethanol	4 g/kg	Ethanol withdrawal is associated with c-Fos expression in the basal ganglia and associated circuitry	[39]
Ethanol	2 g/kg	45 ethanol-related phenotypes were significantly correlated with *Coq7* expression	[41]
Cocaine	60 mg/kg	QTL association on chromosomes 6 and 12 with susceptibility to cocaine-induced seizures	[50]
Cocaine	a	TLs associated with the susceptibility to cocaine-induced seizures on chromosomes 9 (proximal) and 15 (distal) and on chromosome 9 were sex-specific	[51]
Cocaine	b	Genes on proximal chromosome 19 were correlated with DAT expression levels	[54]
Cocaine	5, 10 and 40 mg/kg	QTLs for locomotion on chromosomes 1, 3–6, 9, 12, 13, 18, and 19 were associated with cocaine response	[55]
Iron	240 ppm	Glutamate transporter 1 (GLT1) gene on chromosome 2 as a key regulator gene of iron homeostasis	[63]
Iron	240 ppm	Identified 11 regions with nucleotide polymorphism on chromosomes 1, 2, 5, 7, 9. 11, 13, 14, 17, and 18 in the ventral midbrain	[72]
Iron	240 ppm	BXD 19 and 31 strains had higher up-regulation of Hbb-b1	[75]
Copper	13 ppm	Association between copper regulation and QTL analyses on chromosome 16 in the caudate-putamen (CP) of males, QTL on chromosome 12 in the CP and nucleus accumbens (NA) in females and on chromosomes 3, 13, and 17 in the prefrontal cortex (PFC) of males	[18]
Copper	b	QTL in chromosomes 14 and 9 associated with copper homeostasis	[59]
Zinc	70 ppm	Zn concentration was correlated in QTL region on chromosome 8 in the NA of females and on chromosomes 3, 13, and 17 in males in PFC	[18]
Zinc	b	QTL on chromosome 9 was correlated with zinc content in the hippocampus	[59]
Diisopropyl fluorophosphate	4 mg/kg	21 differentially expressed genes having significant QTLs related to CORT + DFP	[3]
Paraoxon	0–1 mg/kg	Paraoxon treatment reduced locomotor activity and this depression was dependent on the genotype	[96]

a: Doses needed to induce a running, bouncing clonic seizure and a tonic hindlimb extensor seizure were recorded for each mouse. b: not informed.

## Data Availability

Not applicable.
